# Dysdiadochokinesia, Ataxia, and Anemia: A Sign of Intraluminal Malignant Mesothelioma?

**DOI:** 10.14309/crj.0000000000000560

**Published:** 2021-04-27

**Authors:** Raquel Rozner, Shawn L. Shah, Kenrry Chiu, Carl V. Crawford

**Affiliations:** 1 Division of Gastroenterology and Hepatology, New York-Presbyterian Hospital and Weill Cornell Medical Center, New York, NY; 2 Department of Pathology and Laboratory Medicine, Royal Columbian Hospital, New Westminster, Canada

## Abstract

An 87-year-old man presented with altered mental status and ataxia was found to have a neuron-restricted antibody in his cerebrospinal fluid, concerning for a paraneoplastic syndrome of unknown origin. He also exhibited anemia, but otherwise normal electrolytes and liver chemistries. He underwent positron emission tomography/computed tomography which revealed abdominal lymphenopathy. He then underwent push enteroscopy and was found to have a jejunal mass, biopsy proven to be malignant mesothelioma. Malignant mesothelioma is 4–5 times more prevalent in men than women. It is limited to the small bowel, and paraneoplastic syndromes are extremely rare and carry a poor prognosis. The presence of anemia with cerebellar symptoms should trigger a search for a paraneoplastic syndrome-related malignancy.

## INTRODUCTION

Malignant mesothelioma is a rare neoplasm that arises from the mesothelial surfaces of serosal membranes, pleura, and peritoneum, causing significant morbidity and mortality as most patients present with advanced disease.^1^ Although peritoneal mesothelioma can encase and invade intestines, presentation as a discrete intestinal intraluminal mass is unusual. Here, we present a case of an elderly man with dysdiadochokinesia found to have abdominal lymphadenopathy and an intraluminal jejunal mass from a peritoneal mesothelioma with an associated paraneoplastic phenomenon.

## CASE REPORT

An 87-year-old man with a family history of colon cancer in 1 first-degree relative presented with several months of unintentional weight loss, gait instability, and trouble performing rapid, alternating movements. Two months before admission, the patient presented to an outside hospital with chief complaint of 1 week of vertigo, difficulty walking due to balance issues, and slurred speech. At the outside hospital, workup included an abdominal and pelvic computed tomography with contrast that only demonstrated abnormal hyperenhancement along the left posterior aspect of the prostate, which prompted a paraneoplastic evaluation with a magnetic resonance imaging of the brain. It was negative for an intracranial abnormality, but he exhibited a positive cerebrospinal fluid analysis for a neuron-restricted autoantibody, concerning for a paraneoplastic neurologic syndrome (PNS). He was treated empirically with 5 days of intravenous immunoglobulin (IVIG) and 3 days of oral prednisone 60 mg daily, with minimal improvement in symptoms.

**Figure 1.**
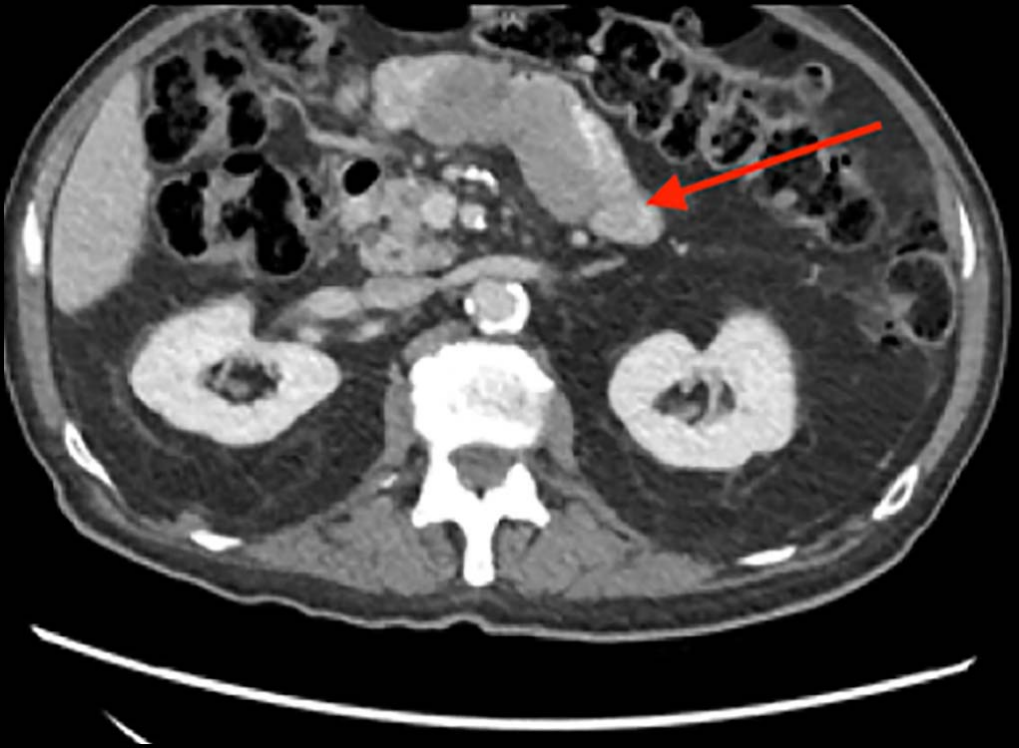
Abdominal and pelvic computed tomography imaging displaying a lymph node inseparable from a loop of proximal small bowel measuring 7.3 × 2.4 cm.

**Figure 2.**
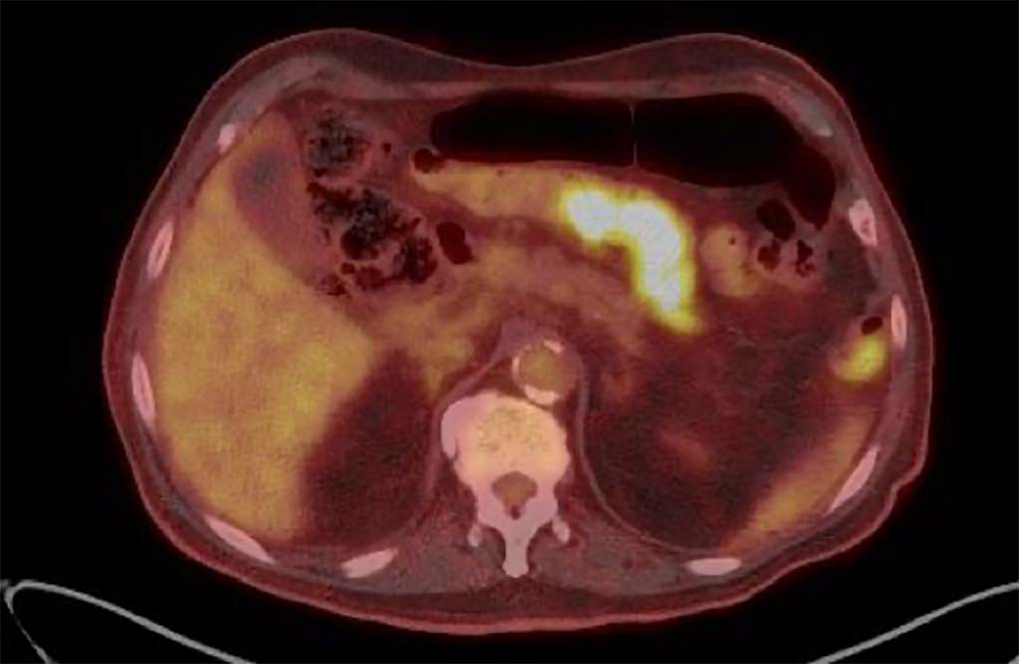
Positron emission tomography/computed tomography showing F-fluorodeoxyglucose avidity of the small bowel.

**Figure 3.**
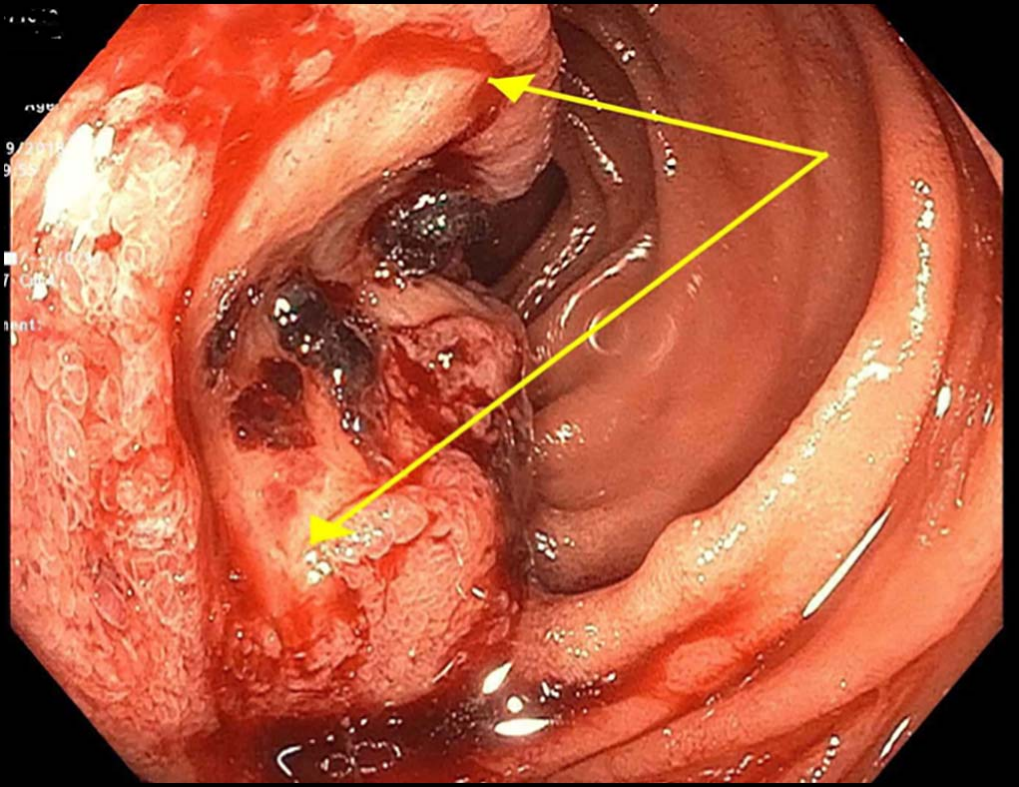
Push enteroscopy identifying an oozing jejunal mass that was consistent with malignant pleural mesothelioma.

**Figure 4.**
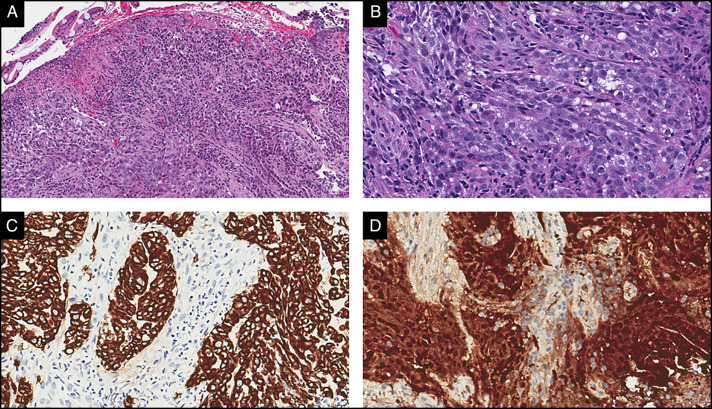
Jejunal biopsy showed (A and B) sheets and nests of epithelioid tumor cells. Immunohistochemical workup showed that the tumor was diffusely positive for (C) pan-cytokeratin and (D) calretinin.

Physical examination was notable for gait ataxia and dysdiadochokinesia with a benign abdominal examination. He had normal electrolytes and liver chemistries, but an elevated prostate-specific antigen of 9.0 ng/mL, decreased hemoglobin of 9.8 g/dL along with low iron level of 17 μg/dL, and low iron saturation of 5.8%. Positron emission tomography/computed tomography revealed intensely F-fluorodeoxyglucose-avid abdominal and mesenteric lymph nodes, one inseparable from the small bowel and increased avidity in the prostate (Figures 1 and 2). As the lymph nodes were not accessible by interventional radiology-guided biopsy, he underwent a push enteroscopy to evaluate the small bowel. On enteroscopy, a large, oozing ulcerated mass was found in the proximal jejunum and biopsied (Figure 3). The biopsy showed sheets and nests of epithelioid tumor cells (Figure 4). Immunohistochemical stains showed that the tumor was strongly and diffusely positive for cytokeratin, CK7, WT-1, and calretinin, patchy weakly positive for D2-40, and negative for NKX3.1, PSMA, and prostate-specific antigen, CK20, CDX2, GATA-3, and TTF-1. These results supported the diagnosis of mesothelioma. His neurologic decline was then attributed to a PNS secondary to peritoneal mesothelioma. He was also found to have prostate cancer. He was discharged with plans to either start chemotherapy or choose hospice care. Unfortunately, he has been lost to follow-up.

## DISCUSSION

Malignant mesothelioma strongly correlates with asbestos exposure and is 4–5 times more prevalent in men than women.^2^ Our case of peritoneal mesothelioma presenting clinically as a discrete small bowel mass is unusual. Peritoneal mesothelioma rarely mimics a visceral tumor radiographically.^3^ PNS affects 1 in 10,000 patients with cancer,^4^ or roughly 1%–3% of all patients with cancers, with more than 85% of cases linked to gynecologic and breast cancers causing cerebellar dysfunction through Purkinje cell destruction by anti-Yo Ab.^5^ Two cases of peritoneal mesothelioma-associated PNS with anti-Yo Ab in the cerebrospinal fluid have been reported; 1 was treated with IVIG and high-dose steroids, while the other refused IVIG and steroids to pursue further chemotherapy.^6,7^ Aside from treating the underlying cancer, stabilizing neurological symptoms with immune modulators are necessary for PNS.^8^

The proposed mechanisms of how IVIG is effective in treating PNS include binding to the Fc receptor on the host's immune cells to prevent them from targeting neurons, neutralizing the autoantibody on neurons, increasing the number of regulatory T cells, and accelerating catabolism of PNS antibodies.^8^ Plasmapheresis is another possible treatment to remove circulating antibodies.^8^ Despite these treatments, the prognosis of peritoneal mesothelioma and associated PNS remains poor. Although exceedingly rare, the presence of anemia with cerebellar symptoms such as dysdiadochokinesia should trigger a search for a PNS-related malignancy including more rare forms such as peritoneal mesothelioma.

## DISCLOSURES

Authors contributions: R. Rozner, SL Shah, and CV Crawford wrote the manuscript and approved the final manuscript. K. Chiu revised the manuscript for intellectual content and approved the final manuscript. CV Crawford is the article guarantor.

Financial disclosure: None to report.

Previous presentation: This case was presented at the American College of Gastroenterology Annual Scientific Meeting; October 25–30, 2019; San Antonio, Texas.

Informed consent was obtained for this case report.

## References

[1] Kannerstein M Churg J. Peritoneal mesothelioma. Hum Pathol 1977;8(1):83–94. 844856 10.1016/s0046-8177(77)80067-1

[2] Carbone M Ly BH Dodson RF. Malignant mesothelioma: Facts, myths, and hypotheses. J Cel Physiol 2012;227(1):44–58. 10.1002/jcp.22724 PMC3143206 21412769

[3] Park JY Kim KW Kwon H. Peritoneal mesotheliomas: Clinicopathologic features, CT findings, and differential diagnosis. AJR Am J Roentgenol 2008;191(3):814–825. 18716115 10.2214/AJR.07.3628

[4] Darnell RB Posner JB. Paraneoplastic syndromes affecting the nervous system. Semin Oncol 2006;33(3):270–98. 16769417 10.1053/j.seminoncol.2006.03.008

[5] Rosenfeld MR Dalmau J. Paraneoplastic neurologic syndromes. Neurol Clin 2018;36(3):675–85. 30072076 10.1016/j.ncl.2018.04.015

[6] Tassinari D Sartori S Arcangeli V. Subacute cerebellar degeneration and pleural mesothelioma. report of a case [in Italian]. Recenti Prog Med 2000;91(6):301–2. 11512388

[7] Tanriverdi O Meydan N Barutca S Ozsan N Gurel D Veral A. Anti-yo antibody-mediated paraneoplastic cerebellar degeneration in a female patient with pleural malignant mesothelioma. Jpn J Clin Oncol 2013;43(5):563–8. 23475537 10.1093/jjco/hyt031

[8] Pelosof LC Gerber DE. Paraneoplastic syndromes: An approach to diagnosis and treatment. Mayo Clin Proc 2010;85(9):838–54. 20810794 10.4065/mcp.2010.0099 PMC2931619

